# Multimodal magnetic resonance imaging in relation to cognitive impairment in neuromyelitis optica spectrum disorder

**DOI:** 10.1038/s41598-017-08889-9

**Published:** 2017-08-23

**Authors:** Su-Hyun Kim, Eun Young Park, Boram Park, Jae-Won Hyun, Na Young Park, AeRan Joung, Sang Hyun Lee, Ho Jin Kim

**Affiliations:** 1 Department of Neurology, Research Institute and Hospital of National Cancer Center, Goyang, Korea; 2 Biometric Research Branch, Research Institute and Hospital of National Cancer Center, Goyang, Korea; 3 Department of Radiology, Research Institute and Hospital of National Cancer Center, Goyang, Korea

## Abstract

Cognitive impairment (CI) is reported in 29–57% of patients with neuromyelitis optica spectrum disorder (NMOSD). However, the pathophysiology underlying CI in NMOSD is poorly understood. The present study aims to investigate the predictive values of various conventional and quantitative MRI parameters for cognitive performance in patients with NMOSD. Neurological assessment and conventional, diffusion tensor, and volumetric MRI sequences were collected form 73 patients with NMOSD and 44 healthy controls (HCs). Patients with ≥3 failed tests were considered to have CI. Brain lesion load, gray matter (GM) and white matter (WM) atrophy, deep GM (DGM) atrophy, cortical thickness, and diffuse microstructural WM damage were assessed. Twenty-three (32%) patients with NMOSD had CI. Compared to cognitively preserved (CP) individuals, patients with CI had atrophy in the WM, thalamus, and caudate, decreased fractional anisotropy (FA) and increased mean diffusivity in their WM. A multivariate model indicated that mean FA values in the WM and volume in the nucleus accumbens (NAc) were associated with overall cognition (p = 0.002 and p = 0.008, respectively). Diffuse microstructural damage in the WM and DGM atrophy in the NAc are the strongest predictors of cognitive impairment in patients with NMOSD.

## Introduction

Neuromyelitis optica spectrum disorder (NMOSD) is a rare inflammatory disease of the central nervous system characterized by attacks of optic neuritis, longitudinally extensive transverse myelitis, or area postrema syndrome^1^. Since the discovery of disease-specific aquaporin-4 (AQP-4) antibodies^2^, the clinical spectrum of NMOSD has been broadened. Cognitive impairment (CI) is reported in 29–57% of patients with NMOSD^3–7^. However, the pathophysiology underlying CI in NMOSD is poorly understood.

Magnetic resonance imaging (MRI) is a powerful and versatile brain imaging modality used to investigate the pathophysiology of neurological disorders *in vivo*. To date, few studies have attempted to find the relevant MRI substrate of CI in NMOSD. This has led to conflicting results. One study found that global and focal white matter (WM) atrophy, but not gray matter (GM) atrophy, is correlated with cognitive impairment^8^. On the other hand, GM volume reduction in the medial prefrontal cortex and the thalamus predict impairments in memory, information processing speed, and verbal fluency^9^. Another study from China reported that microstructural changes in the corpus callosum play a role in cognitive impairment during acute relapse in NMOSD^10^. Nevertheless, most of the above studies used a single MRI modality or evaluated confined brain regions. Furthermore, they mainly investigated patients without brain lesions on MRI. These groups might not represent the full spectrum of NMOSD, as brain MRI abnormalities have been reported in 50–89% of patients with NMOSD^11^. The variable results of previous MRI studies imply the presence of a complex substrate for cognitive impairment in NMOSD and emphasize the need for more comprehensive approaches using multiple different MRI modalities.

We aimed to identify the strongest MRI predictors of cognitive impairment in NMOSD. We used different structural MRI modalities to define the relative contributions of lesion load, global atrophy, cortical thickness, normal appearing WM (NAWM) damage, and deep GM damage to cognitive deficits and impairments of specific cognitive domains in patients with NMOSD.

## Results

### Demographic and clinical characteristics

Seventy-three patients with NMOSD and 44 HCs were included in our study. Sixty-five (89%) patients were positive for anti-AQP4 antibody. No differences in age, gender, or educational level were found between patients with NMOSD and the HCs, but patients with NMOSD differed from the HCs in measures of depression (Table 1). Twenty-three (32%) of the 73 patients were classified as CI. Age, disease duration, time from onset to treatment, EDSS score, seropositivity for anti-AQP4 antibody, and frequency of patients with symptomatic medications were comparable between CP patients and those with CI. Patients with CI had lower education levels and higher depression scores than CP patients.

**Table 1:** Demographic and clinical characteristics of healthy controls, patients with NMOSD, and the cognitively preserved and impaired patient groups.

	Healthy controls (n = 44)	Patients with NMOSD (n = 73)	Cognitively preserved patients (n = 50)	Patients with cognitive impairment (n = 23)
Age, y	38.6 ± 6.8	36.8 ± 6.9	35.8 ± 6.5	39.0 ± 7.4
Gender, F/M	39/5	70/3	47/3	23/0
Education, years, mean ± SD	15 ± 2	15 ± 2	15 ± 2	14 ± 2^*,##^
PHQ-9 score, mean ± SD	4 ± 3	10 ± 8^***^	7 ± 5	13 ± 10^***,###^
Anti-aquaporin4 antibody, n (%)	NA	65 (89%)	45 (90%)	20 (87%)
Disease duration, years, mean ± SD	NA	7.8 ± 4.3	7.6 ± 4.1	8.1 ± 5.0
Time to treatment, years, mean ± SD	NA	3.4 ± 3.5	3.1 ± 3.2	3.9 ± 4.2
Median EDSS score (range)	NA	2.0 (0–7.0)	2.0 (0–7.0)	3.0 (0–7.0)
Mean VF score, mean ± SD	NA	0.9 (1.3)	0.8 (1.2)	1.1 (1.5)
Symptomatic medication for neuropathic pain, n (%)	NA	14 (19%)	10 (20%)	4 (18%)
Immunosuppressive treatment, n (%)	NA	AZA 7 (9%) MMF 24 (33%) Rituximab 42 (58%)	AZA 6 (12%) MMF 15 (30%) Rituximab 29 (58%)	AZA 1 (4%) MMF 9 (39%) Rituximab 13 (57%)

Abbreviations: BFI = Brief Fatigue Inventory; EDSS = Expanded Disability Status Scale; HCs = healthy controls; NA = not applicable; NMOSD = neuromyelitis optica spectrum disorder; PHQ-9 = Patient Health Questionnaire-9; SD = standard deviation; AZA = azathioprine; MMF = mycophenolate mofetil. Post hoc analysis was performed using Bonferroni correction.

^*^p < 0.05 compared to healthy controls.

^**^p < 0.01 compared to healthy controls.

^***^p < 0.001 compared to healthy controls.

^#^p < 0.05 compared to cognitively preserved patients.

^##^p < 0.05 compared to cognitively preserved patients.

^###^p < 0.05 compared to cognitively preserved patients.

### Cognition

Patients with NMOSD had significantly impaired performance in visual memory (immediate and delayed recall), SDMT, backward Digit-Span, Stroop interference, PASAT, and global cognitive score (z-scores) compared to HCs (Table 2). Patients with CI had worse performance on all cognitive domains compared to CP patients (Table 3).

**Table 2:** Comparison of cognitive scores of healthy controls and patients with NMOSD.

Cognitive tests, adjusted mean scores (SE)	Healthy controls (n = 44)	Patients with NMOSD (n = 73)
Verbal memory		
immediate recall	−0.51 (0.21)	−0.73 (0.15)
delayed recall	−0.38 (0.2)	−0.55 (0.14)
recognition	−0.43 (0.31)	−0.55 (0.24)
Visuospatial function		
RCFT copy score	−0.15 (0.25)	−0.54 (0.18)
Visual memory		
immediate recall	−0.48 (0.25)	−1.08 (0.18)^*^
delayed recall	−0.46 (0.26)	−1.24 (0.18)^**^
K−WAIS symbol digit modalities test	−0.52 (0.2)	−1.05 (0.14)^*^
Digit Span		
forward	−0.3 (0.21)	−0.28 (0.15)
backward	−0.04 (0.17)	−0.54 (0.12)^**^
Stroop test		
simple	−0.33 (0.24)	−0.68 (0.17)
interference	−0.4 (0.27)	−1.2 (0.19)^**^
COWAT	−0.58 (0.21)	−0.81 (0.15)
PASAT	−0.72 (0.31)	−1.54 (0.22)^*^
Global z−score (sum of subtest scores)	−5.3 (1.85)	−10.78 (1.31)^**^

Abbreviations: BFI = Brief Fatigue Inventory; EDSS = Expanded Disability Status Scale; HCs = healthy controls; NA = not applicable; NMOSD = neuromyelitis optica spectrum disorder; PHQ-9 = Patient Health Questionnaire-9; SD = standard deviation; SE = standard error. All comparisons of cognitive function were corrected for age, educational level, and depression level.

^*^p < 0.05 compared to healthy controls.

^**^p < 0.01 compared to healthy controls.

**Table 3:** Comparison of cognitive scores of healthy controls, cognitively preserved, and impaired patient groups.

Cognitive tests, adjusted mean scores (SE)	Healthy controls (n = 44)	Cognitively preserved patients (n = 50)	Patients with cognitive impairment (n = 23)
Verbal memory			
immediate recall	−0.38 (0.21)	−0.33 (0.18)	−1.36 (0.22)^**,##^
delayed recall	−0.27 (0.19)	−0.19 (0.17)	−1.11 (0.21)^**,###^
recognition	−0.28 (0.3)	−0.07 (0.26)	−1.32 (0.32)^##^
Visuospatial function			
RCFT copy score	−0.01 (0.24)	−0.07 (0.21)	−1.29 (0.26)^**,##^
Visual memory			
immediate recall	−0.21 (0.21)	−0.21 (0.18)	−2.45 (0.22)^***,###^
delayed recall	−0.19 (0.21)	−0.35 (0.19)	−2.61 (0.23)^***,###^
K−WAIS symbol digit modalities test	−0.41 (0.19)	−0.69 (0.17)	−1.62 (0.21)^***,##^
Digit Span			
forward	−0.19 (0.2)	0.07 (0.17)	−0.84 (0.22)^##^
backward	0.03 (0.17)	−0.3 (0.15)	−0.92 (0.18)^**,#^
Stroop test			
simple	−0.22 (0.23)	−0.3 (0.2)	−1.26 (0.25)^**,##^
interference	−0.24 (0.26)	−0.67 (0.23)	−2.03 (0.28)^***,##^
COWAT	−0.42 (0.2)	−0.28 (0.17)	−1.65 (0.21)^***,###^
PASAT	−0.43 (0.26)	−0.6 (0.23)	−3.02 (0.29)^***,###^
Global z−score (sum of subtest scores)	−3.21 (1.44)	−3.99 (1.25)	−21.49 (1.56)^***,###^

Abbreviations: BFI = Brief Fatigue Inventory; EDSS = Expanded Disability Status Scale; HCs = healthy controls; NA = not applicable; NMOSD = neuromyelitis optica spectrum disorder; SE = standard error. All comparisons of cognitive function were corrected for age, educational level, and depression level. Post hoc analysis was performed using Bonferroni correction.

^*^p < 0.05 compared to healthy controls.

^**^p < 0.01 compared to healthy controls.

^***^p < 0.001 compared to healthy controls.

^#^p < 0.05 compared to cognitively preserved patients.

^##^p < 0.01 compared to cognitively preserved patients.

^###^p < 0.001 compared to cognitively preserved patients.

### Comparisons of individual MRI parameters

Of the 73 patients with NMOSD, 34 (46%) had no brain lesions and 39 (54%) had chronic brain lesions characteristic of NMOSD or small non-specific WM lesions. Patients with NMOSD had lower GMF (p = 0.047), BPF (p = 0.005), mean cortical thickness (p < 0.001), and mean FA values of NAWM (p < 0.001) than HCs. They also had higher mean MD and RD values of NAWM (p < 0.001 and p < 0.001, respectively). Patients with CI had lower WMF and smaller volumes in the thalamus and caudate than CP individuals (Table 4). They also had diffuse changes in NAWM (lower FA and higher MD and RD than CP patients). No significant differences in the volume of hyperintense lesions on FLAIR images or cortical thickness were detected between patients with CI and CP individuals.

**Table 4:** MRI measures of healthy controls, the cognitively preserved and cognitively impaired patient groups.

Adjusted mean scores (SE)	Healthy controls (n = 44)	Cognitively preserved patients (n = 50)	Patients with cognitive impairment (n = 23)
BPF	0.88 (0.003)	0.88 (0.003)	0.87 (0.004)^**^
GMF	0.51 (0.004)	0.49 (0.004)^*^	0.51 (0.006)
WMF	0.37 (0.004)	0.38 (0.004)	0.36 (0.006)^##^
FHLV, ml	NA	5.5 (0.8)^*^	6.8 (1.2)^*^
Cortical thickness, mm	3.02 (0.02)	2.88 (0.02)^***^	2.86 (0.03)^***^
Frontal	3.11 (0.03)	2.94 (0.03)^***^	2.92 (0.04)^**^
Parietal	2.92 (0.03)	2.77 (0.02)^***^	2.73 (0.04)^***^
Temporal	3.37 (0.03)	3.19 (0.03)^***^	3.17 (0.05)^**^
Occipital	2.75 (0.02)	2.61 (0.02)^***^	2.62 (0.03)^*^
Deep gray matter volume (10^3^ ml)			
Thalamus	9.9 (0.14)	9.85 (0.13)	9.17 (0.19)^**,#^
Putamen	5.98 (0.09)	5.9 (0.09)	5.64 (0.13)
Pallidum	2.23 (0.07)	2.3 (0.07)	2.25 (0.1)
Hippocampus	4.73 (0.09)	4.89 (0.09)	4.58 (0.13)
Accumbens	5.2 (0.2)	5.16 (0.19)	4.42 (0.28)
Amygdala	1.4 (0.04)	1.38 (0.04)	1.4 (0.05)
Caudate	4.64 (0.09)	4.88 (0.09)	4.47 (0.13)^#^
DTI: whole NAWM skeleton			
Mean FA (10^–1^)	5.21 (0.04)	4.96 (0.04)^***^	4.75 (0.06)^***,#^
Mean MD (10^–4^ mm^2^/s)	7.89 (0.06)	8.09 (0.06)	8.45 (0.09)^***,##^
Mean AD (10^–4^ mm^2^/s)	12.72 (0.06)	12.81 (0.06)	12.99 (0.08)^*^
Mean RD (10^–4^ mm^2^/s)	5.49 (0.08)	5.77 (0.07)^*^	6.25 (0.11) ^***,##^

Abbreviations: BPF = brain parenchymal fraction; FHLV = FLAIR-hyperintense lesion volume; GMF = gray matter fraction; HCs = healthy controls; NA = not applicable; NMOSD = neuromyelitis optica spectrum disorder; SD = standard deviation; SE = standard error; WMF = white matter fraction. All comparisons were corrected for age. Post hoc analysis was performed using Bonferroni correction.

^*^p < 0.05 compared to healthy controls.

^**^p < 0.01 compared to healthy controls.

^***^p < 0.001 compared to healthy controls.

^#^p < 0.05 compared to cognitively preserved patients.

^##^p < 0.01 compared to cognitively preserved patients.

### Association analyses

In patients with NMOSD, global cognitive z-score associated with low education (p < 0.001) and depression (p = 0.005). Old age negatively associated with delayed recall of visuospatial memory (p = 0.049), SDMT score (p = 0.014), and PASAT score (p = 0.012). EDSS scores negatively associated with immediate recall of visuospatial memory, PASAT, and global cognitive z-score (p = 0.036, p = 0.018, and p = 0.017, respectively). Global cognitive z-scores positively associated with mean FA values (p < 0.001), WMF (p = 0.037), BPF (p = 0.018), and volumes of thalamus (p = 0.006), NAc (p < 0.001) and caudate (p = 0.006), and negatively associated with mean MD values (p = 0.005). Immediate recall and delayed recall of visual memory positively associated with mean FA values (p < 0.001 and p < 0.001, respectively), volumes of thalamus (p < 0.001 and p = 0.015, respectively), putamen (p < 0.001 and p = 0.012, respectively), and caudate (p = 0.002 and p = 0.014, respectively), and negatively associated with mean MD values (p < 0.001 and p = 0.004, respectively). Hippocampal atrophy was associated with poor performance in immediate recall of visual memory (p = 0.006). SDMT scores positively associated with mean FA values (p = 0.004), BPF (p = 0.044), and volumes of thalamus (0.045), putamen (0.015), NAc (p = 0.01), and caudate (p = 0.005). Finally, PASAT scores were positively associated with mean FA values (p = 0.027) and NAc volume (p = 0.01). We found no associations between the hyperintense lesion volume on FLAIR images and cortical thickness or cognitive performance.

### Multivariate models

Table 5 summarizes the results of the multivariate regression analysis performed to identify MRI variables significantly associated with overall global cognitive z-scores and performance in specific cognitive domains. Based on comparisons between CP patients and those with CI and univariate correlate analyses, different candidate predictors for multivariate analyses were used for each cognitive function such as WMF, DGM volume (in the thalamus, putamen, NAc, hippocampus, and caudate), FA, and MD in NAWM. A multivariate model indicated that FA of NAWM and accumbens atrophy were independently associated with global cognitive z-scores (p = 0.002 and p = 0.008, respectively). NAc atrophy was associated with performance on the PASAT (p = 0.007) and immediate recall of visual memory (p = 0.028). FA abnormalities were associated with worse performance in immediate recall (p ≤ 0.001) and delayed recall (p < 0.001) of visuospatial memory and SDMT (p = 0.004). A weak associated was also observed between caudate atrophy and performance on the Stroop interference test (p = 0.018).

**Table 5:** Multivariate regression analysis to identify MRI variables associated with cognition.

	Global z-score		Immediate recall of visual memory		Delayed recall of visual memory		SDMT		Stoop-interferen		PASAT	
	Beta (95%CI)	P value	Beta (95%CI)	P value	Beta (95%CI)	P value	Beta (95%CI)	P value	Beta (95%CI)	P value	Beta (95%CI)	P value
Mean FA in NAWM	12.34 (4.82, 19.87)	0.002	1.95 (0.97, 2.94)	< 0.001	2.0 (0.96, 3.04)	< 0.001	1.19 (0.4, 1.98)	0.004				
Nucleus Accumbens	2.59 (0.68, 4.49)	0.008	0.28 (0.03, 0.53)	0.028							0.43 (0.12,0.74)	0.007
Caudate									0.69 (0.12, 1.26)	0.018		

Abbreviations: FA = fractional anisotropy; NAWM = normal appearing white matter; PASAT = Paced Auditory Serial Addition Test; SDMT = symbol digit modalities test; 95%CI = 95% confidence interval.

## Discussion

By combining voxel-wise analysis methods and a multiparametric structural MRI approach, which included measures of both irreversible tissue loss and microstructural tissue damage, we show that CI in NMOSD is the result of a complex interplay between NAWM and GM damage. We found that 32% of patients with NMOSD have CI, with core deficits in attention/information processing speed and visuospatial episodic memory despite preserved verbal episodic memory and verbal fluency. In patients with NMOSD, diffuse white matter microstructural damage (mean FA value in NAWM) and DGM (especially NAc) atrophy are the strongest predictors for CI. Conversely, brain lesion load, total brain volume, and cortical thickness were not associated with CI. This suggests that the role of conventional imaging measures in assessing cognitive impairment is limited.

We have previously reported a lower prevalence of CI and different patterns of CI in patients with NMOSD compared to patients with multiple sclerosis (MS). Specifically, patients with MS have more widespread CI with greater impairment, particularly in verbal episodic memory^4^. CI correlates with lesion burden, brain atrophy, and cortical thickness in patients with MS, while these MRI parameters are not significantly associated with cognitive function in patients with NMOSD^4, 12^. The differences in the frequency and pattern of CI and the different MRI substrates of CI between the two diseases might suggest different pathophysiologies of brain injury in the two diseases. Several studies of patients with MS have attempted to identify relevant MRI substrates using multiparametric structural MRI approaches. Diffuse WM microstructural damage and deep gray matter atrophy, particularly in the thalamus and hippocampus, are consistently reported as strong predictors of cognitive impairment in MS^13–15^. We found that diffuse WM damage also contributes to CI in NMOSD. Nevertheless, observation of diffuse WM damage, even in CP patients, suggests that CI is not evident until a certain threshold level of WM damage occurs. It is worth noting that we found different patterns of subcortical atrophy in patients with NMOSD compared to previous studies of patients with MS. For instance, NAc atrophy was prominently correlated in NMOSD patients with CI. The associations between thalamic, WM or global brain atrophy, and cognition in the univariate analysis were consistent with the results of previous studies of NMOSD^8, 9^. However, these associations were not significant in the multivariate analysis of the current study. These findings suggest that the use of a single MRI modality is not sufficient to properly explain cognitive impairment in NMOSD. Recently, Liu and colleagues suggested that hippocampal atrophy is the main MRI predictor of cognition in NMOSD using a multiparametrical MRI approach^7^. However, we found no significant correlations between hippocampal atrophy and CI. No relationships between brain lesion load or cortical thickness and cognition in patients with NMOSD in the current study were consistent with Liu’s study^7^. The discrepancies may be explained by differences in the clinical characteristics of the patients enrolled, the methods used for neuropsychological evaluation, and the statistical analysis methods.

The discovery of a relationship between NAc atrophy and cognition in patients with NMOSD is novel and warrants further consideration. AQP4 expression has been shown to be higher in the hypothalamic and periventricular areas of the brain^16^. The NAc is a region in the basal forebrain rostral to the preoptic area of the hypothalamus and has important functional relationships with the hypothalamus^17^. The NAc’s location may make it more susceptible to NMOSD-related brain injury. NAc plays a central functional role in positive emotional responses. In fact, patients with mood disorders, such as depression, have NAc atrophy^17^. In our cohort, patients with CI had higher depression scores than CP patients. Consistently, a recent study indicates that 28% of patients with NMOSD suffer from unrecognized depression and that the severity of depression is not associated with physical disability or disease duration^18^. Furthermore, the NAc is involved in the generation of motivated behavior and NAc atrophy is suggested to underlie the apathy of patients with Parkinson’s disease^19^. Thus it is conceivable that the decrease in desire induced by NAc atrophy might also contribute to the impairment observed in attention/information processing speed in patients with NMOSD.

Our study has limitations. First of all, this study was a single-center study, which may have introduced selection bias when compared to a population-wide study. We selected a priori a set of imaging modalities (3D T1-weighted and DTI) and thus cannot rule out that the use of other imaging modalities (e.g., myelin water imaging or functional MRI) would have led to different results. In addition, as there is no neuropsychological battery developed specifically for NMOSD, evaluation using the brief battery in the current study may limit the comprehensive evaluation of CI. As our neuropsychological battery has not been validated elsewhere, direct comparison of our results with other studies warrants caution. Finally, NMOSD pathology in this study was not directly observed; inferences about these pathologies were based on MRI findings. Future research will be necessary to verify or refute our hypothesis of MRI findings corresponding to the underlying pathologies. Additionally, future research is needed to clarify the relationships of longitudinal MRI changes with cognitive decline. The development and validation of useful MRI biomarkers for CI will enable MRI to be utilized as a powerful tool for the evaluation of therapeutic efficacy in NMOSD clinical trials.

## Methods

### Patients

Seventy-three patients with NMOSD were recruited prospectively from the Department of Neurology of the National Cancer Center (NCC) in Korea between December 2013 and June 2015. NMOSD was diagnosed according to the 2006 Criteria for NMOSD^20^ or one of the cardinal symptoms of NMO with seropositivity for anti-AQP4 antibodies^21^. Eligible individuals were aged 20–50 years, relapse- and steroid-free for at least 6 months, and had undergone both, MRI and neuropsychological tests. Exclusion criteria included co-morbid neurological diseases other than NMOSD, alcohol or other substance abuse, and a history of psychiatric illness, except for stable depressive symptoms. Forty-four age- and sex-matched healthy controls (HCs) without a history of neurological or psychiatric illness or substance abuse were recruited. The following parameters were investigated: current age, gender, disease duration, time interval from disease onset to treatment initiation, anti-AQP4 antibody status, and disability, as measured by the Expanded Disability Status Scale (EDSS). This study was approved by the Institutional Review Board of the NCC. Written informed consent was obtained from all patients. All experiments were performed in accordance with relevant guidelines and regulations.

### Neuropsychological assessment

All patients with NMOSD and HCs underwent neuropsychological tests to assess cognitive function. The neuropsychological battery comprised tests for verbal/visual memory, visuospatial function, attention, information processing speed, language, and working memory^4^. Verbal learning and memory were assessed using the Seoul Verbal Learning Test (SVLT)^22^ and the Korean version of the Hopkins Verbal Learning Test-revised (HVLT-R)^23^. The visual memory score was composed of scores from the immediate recall and delayed recall portions of the Rey Complex Figure Test (RCFT)^24^. The Controlled Oral Word Association Test (COWAT)^25^ was administered to assess associative verbal fluency for phonemic stimuli. The Symbol Digit Modalities Test (SDMT)^26^, the Paced Auditory Serial Addition Test (PASAT)^27^ at 3 seconds and Stroop Color and Word tests^28^ were used to assess sustained attention and the speed of information processing. In addition, the Digit Span^29^ test was administered to assess working memory^30^. Each subject completed a questionnaire regarding depressive symptoms (Patient Health Questionnaire-9, PHQ-9). Because depression itself negatively impacts cognitive performance, each patient’s raw score was compared to the mean value obtained from HCs matched for PHQ-9 level using z-scores. The z-scores were calculated for each neuropsychological score using the following formula: (patient’s score – mean value from HCs matched for PHQ-9/standard deviation from the matched HCs). The z-scores for specific cognitive domains were combined to generate a global cognitive z-score. Patients were considered to have CI if they scored lower than the fifth percentile of the HCs in at least three domains^4^. The other patients were classified as CP.

### Image acquisition

Imaging was performed using a 3.0-Tesla MR scanner (Philips Achieva; Philips Medical Systems, Best, Netherlands). Brain MRI included structural 3-dimensional T1-weighted scans for volumetric and cortical thickness analysis, axial 2-dimensional T2-weighted and fluid attenuated inversion recovery (FLAIR) scans for lesion detection, and 32-direction diffusion tensor imaging (DTI). The T2, T1, FLAIR, and DT images were acquired using the following sequences: (1) three-dimensional (3D) high-resolution sagittal FLAIR T2-weighted sequence (repetition time [TR] = 4,800 ms, echo time [TE] = 265 ms, inversion time = 1,650 ms, echo train length = 175, field of view [FOV] = 230 mm, matrix = 230 × 230, and voxel size = 1 × 1 × 1 mm); (2) 3D high-resolution T1-weighted sequence (TR = 9.8 ms, TE = 4.5 ms, inversion time = 1,650 ms, flip angle = 8°, FOV = 230 mm, matrix = 230 × 230, slice thickness = 0.5 mm, no gap, and voxel size = 1 × 1 × 0.5 mm); (3) spin-echo single-shot echo planar imaging DTI sequence (TR = 9,500 ms, TE = 75 ms, number of excitations = 1, matrix = 128 × 128, FOV = 230 × 230 mm, number of slices = 80, slice thickness = 2 mm, slice gap = 0 mm, orientation = axial, 32 nonlinear diffusion weighting gradient directions with b = 1,000 s/mm^2^ and one additional b0-volume).

### MRI data analysis

All post-processing of structural MR images was performed at the Department of Biomedical Engineering at Hanyang University in Korea. Images were processed using the standard Montreal Neurological Institute (MNI) pipeline.

### Volumetrics

The 3D T1 images were corrected for intensity non-uniformity artifacts^31^ and normalized to the MNI 152 template using linear transformation^32^. An artificial neural network classifier was used to identify GM, WM, and cerebrospinal fluid. To avoid mistakenly counting lesions as GM volume, we calculated corrected GM volumes and WM volumes as follows: corrected GM volume = uncorrected GM volume - lesion volume in WM, and corrected WM volume = uncorrected WM volume + lesion volume in WM. We divided brain volume by total intracranial volume (ICV) to adjust head size differences. Gray matter fraction (GMF) and white matter fraction (WMF) were calculated as follows: GMF = corrected GM volume/ICV, and WMF = corrected WM volume/ICV, where ICV = brain parenchymal volume + cerebrospinal fluid volume^33^.

After performing an intensity inhomogeneity correction, the images were segmented into 14 DGM structures, including the bilateral nucleus accumbens (NAc), amygdala, caudate, hippocampus, pallidum, putamen, and thalamus using the Oxford Centre for Functional MRI of the Brain (FMRIB) Integrated Registration and Segmentation Tool (FIRST)^34^. The shapes and appearances of the FIRST models were determined based on point distributions through the parameterization of surface meshes built from labels of manually segmented images. Intensity information along each vertex of the surface was also modeled. The models are used to search for optimal modes of shape variation in a novel image by estimating proper Bayesian probabilities. Each voxel in the novel image is assigned an appropriate label. Using the labeled image, the DGM volume corresponding to the label is calculated. The right and left volumes for each DGM structure were averaged and normalized in SIENAX by V-scaling, which consists of calculating the ratio of the subject’s head size to a standard head size to reduce the effects of head size differences between subjects^35^. In order to perform V-scaling, the brain tissue of the subject is first extracted from the subject’s raw image. The brain and skull images are then used to perform V-scaling between the subject’s image and the standard space.

### Lesion load

To analyze the volumes of hyperintense lesions on T2-weighed images, FLAIR images were examined using a previously described thresholding technique^36^. The results of all automated segmentations were visually inspected by a rater experienced in anatomy and volumetry. When necessary, the segmentation was manually corrected using ITK-Snap (www.itksnap.org).

### Cortical thickness

The surfaces of the inner and outer cortices were extracted automatically using the Constrained Laplacian-based Automated Segmentation with Proximities algorithm^32, 37^. Cortical thickness was defined as the Euclidean distance between the linked vertices of the inner and outer surfaces.

### DTI data processing

DTI processing was performed using FMRIB Software Library (FSL) ver. 5.0.2.1 (http://www.fmrib.ox.ac.uk/fsl). Voxel-wise statistical analyses of fractional anisotropy (FA), mean diffusivity (MD), axial diffusivity (AD), and radial diffusivity (RD) were carried out using Tract-Based Spatial Statistics (TBSS)^38^. In order to specifically explore the contribution of microstructural NAWM damage, individual lesion masks after normalization to the FA skeleton space were averaged for each group to obtain lesion probability maps. The analyses included only those WM voxels where the lesion probability was <5%, as the voxels with a lesion probability ≥5% were set to zero^39^. We extracted mean FA, MD, RD, and AD values for global NAWM in each subject.

### Statistical analysis

To compare the clinical characteristics of the different groups, analyses of variance (ANOVAs) was used for continuous variables, and chi-squared tests or Fisher’s exact tests was used for categorical variables. Generalized linear regression was used to assess group differences in cognitive function and MRI variables. To screen for correlates of cognitive function, univariate regression analyses were performed between cognition and clinical characteristics and MRI parameters. These analyses were corrected for age, education level, and depression. Multivariate linear regression analyses were performed to examine associations between MRI parameters and cognitive performance, controlling for age, education level, and depression. MRI variables that were significantly different between CP patients and those with CI or those that correlated with cognition in univariate regression analyses were entered into the multivariate regression analysis. Final multivariate model was determined using backward selection method with elimination criterion of p-value > 0.05. Bonferroni corrections were applied where applicable and p-values < 0.05 were considered statistically significant. SAS (ver. 9.3; SAS Institute, Cary, NC, USA) was used for all analyses.
